# Notes upon a Visit to New York, Philadelphia and Baltimore

**Published:** 1897-06

**Authors:** E. C. Davis

**Affiliations:** Atlanta, Ga.


### NOTES UPON A VISIT TO NEW YORK, PHILADELPHIA AND BALTIMORE.

ATLANTA, GA., April 15, 1897.

Before leaving New York my attention was called by Dr. Robert T. Morris to a series of experiments which he was conducting to prevent an artificial menopause following the removal of fallopian tubes and ovaries for diseased conditions. His method is by a transplantation of healthy ovarian tissue from one patient to another. The plan usually adopted is about as follows: Having two cases requiring the removal of ovaries, in which one has a certain amount of *healthy* ovarian tissue, this is carefully dissected out and preserved in a warm saline solution until the second case is operated upon, when this ovarian tissue is carefully imbedded in the fundus uteri, and in some of the cases has become active in its functions, for menstruation continued, and in one case pregnancy followed, and in another under observation at the present time all indications point to a pregnant condition. Some other observer has conducted a similar series of experiments on the lower animals and reports equally favorable results.

Dr. Morris's simplicity of technique is quite an attractive feature of his surgical work. I witnessed him perform a vaginal hysterectomy in which the instruments used were about as follows: One small knife, a pair of scissors, blunt dissector, two pairs of hemostatic forceps, one pair of long forceps resembling Jacobs's in structure, needle, catgut, and usual dressings. This simplicity applies to all the surgical work witnessed in which he was the operator. Another feature of his work was the apparent confidence in hydrogen dioxide as a destructive agent for pus, and his lack of fear when this infectious agent found its way into the peritoneal cavity, believing that immediate contact with hydrogen dioxide neutralized and rendered innocuous the purulent discharge, or, as he expressed it, the pus was blown out by the peroxide.

Dr. W. R. Pryor should be seen by those visiting the great metropolis, and especially in the performance of a vaginal hysterectomy by morcellation for a large fibroid. In this operation he bisects the uterus after making strong traction to prevent hemorrhage; this tension is persisted in until the operation is completed, and the small amount of blood lost is surprising. As a cleansing agent for the hands he esteems lysol very highly, following its use with Thiersch's solution.

New York abounds with interesting work, and on leaving, one fears lest he will not find such fruitful fields for observation, but on reaching Philadelphia I was agreeably pleased to find an equal activity among surgeons and gynecologists there. Philadelphia can truly be called the city of homes and hospitals, for here the quietude of the residence streets gives a homelike appearance, and the hospitals are very numerous. While walking down Broad street my attention was directed to a sign reading as follows: "Bicycle Hospital—Bicycles treated here without regard to sex, color, or nationality." This is mentioned to show how the hospital idea has taken hold of the city. While on the subject of hospitals I'll mention what is adopted at the German Hospital to protect against infection. This hospital is equipped with a complete pathological and bacteriological laboratory, and before the operations are begun the scrapings from the hands of all who are to handle anything about the operations are inoculated in suitable culture tubes and these placed in the incubator. Each tube is marked so that a record of the nurse, assistant or surgeon from whom obtained can be kept, and if the culture shows in any of the tubes pyogenic micro-organisms this person is debarred from further association with operative work for a specified time. Dr. John B. Deaver is the surgeon for this hospital, and on one afternoon he showed me three operations for appendicitis. The smallness of his incision and the rapidity of his executions make quite a favorable impression upon the observer. He also confines his patients to bed for a much shorter time than is generally adopted in such cases.

Dr. Joseph Price in his private hospital showed his plan of removing uterus by the use of the serre-nœud, leaving the cervix. He also emphasized the frequency of diseased tubes and ovaries in fibromata and the difficulty or impossibility of completely removing such tumors by morcellation, the plan now so popular with many of New York's gynecologists. He too adheres to the great simplicity of technique in his operations and relies upon soap and mustard with plenty of friction from a clean nail brush in cleansing the hands. The Medico-Chirurgical College is just completing a most excellent amphitheater for operative work. This, when completed, will be one of the best to be found anywhere.

Dr. Charles P. Noble, at Kensington Hospital, demonstrated his modification of Emmet's operation on the perineum in quite a number of cases. His plan differs from Dr. Emmet's chiefly in a more thorough dissection of the tissues, and more complete exposure of the levator ani muscle, which is sutured accurately together. He was also greatly interested in the changes he expects to make in Kensington Hospital which, when completed, will render this practically a new place.

Arriving in Baltimore I soon found my way to the Johns Hopkins Hospital where the courteous attention of Dr. H. M. Hurd, superintendent of the hospital, soon makes one feel perfectly at home among such excellent opportunities for study and observation. I often wondered why all who came away from this hospital seemed so greatly impressed with what was seen, but a visit soon makes clear the reasons,—the uniform attention, the absence of unnecessary formality, and the geniality of the professors and others connected with the excellent institution. But this is not intended as a letter of laudation specially, but rather a chronicle of a few observations made. Among the interesting operations witnessed there was one performed by Dr. Thomas S. Cullen, which was the removal of a cyst from the abdominal wall, the removal of a large suppurating ovarian cyst, and the removal of the uterus, all from the same patient. This operation consumed about four hours, and the patient was put to bed in fair condition and no drainage used. The adhesions were very extensive and the trauma necessary to break them up great, yet no drainage was employed. In the surgical department, Dr. Finney opened and washed out the knee joint for a synovitis supposed to be gonorrheal in origin; the other joint had been previously operated upon with very beneficial results. Another case of interest was an epithelioma of lower lip with an involvement of the glands of the neck. The diseased glands of the neck were first removed thoroughly through quite a long incision and very careful dissection, then a V-shaped incision removing the malignant growth was employed.

The operative treatment for some of the cases of cirrhosis of liver was of great interest to me, for beneficial results were said to have followed in a few cases. This plan consisted of the performance of an exploratory incision, with thorough cleansing of the peritoneum.

Dr. Osler's clinic proved very interesting, and especially the report of one case which came under his observation previously and died from tuberculosis, yet repeated microscopic examinations failed to find the tubercle bacillus. This clinic was to me one of the most interesting features of my entire trip, and the special feature of interest was that he made his students do most of the work, and he presided over their work, offering suggestions when they were needed, and criticising certain parts of the reports of cases and examinations which needed criticism. E. C. DAVIS, M.D.

---

THERE is no such thing as "growing pains," says Dr. Irving S. Haynes, in New York State *Medical Reporter*. They mean mischief, and the doctor who uses such a phrase is either too ignorant or too lazy to find out their cause. They mean Pott's disease, and the doctor does not find it out until the limp or the hump in the back appears.—*Ex.*

